# Prevalence of OSA Risk and Bruxism in Children With Autism Spectrum Disorders

**DOI:** 10.1002/aur.70149

**Published:** 2025-11-19

**Authors:** Anna Alessandri‐Bonetti, Federica Guglielmi, Andrea Faustini, Linda Sangalli, Edoardo Staderini, Patrizia Gallenzi

**Affiliations:** ^1^ Institute of Dental Clinic, A. Gemelli University Policlinic IRCCS Catholic University of Sacred Heart Rome Italy; ^2^ Postgraduate School of Orthodontics, Director: Prof. Massimo Cordaro Catholic University of Sacred Heart Rome Italy; ^3^ Postgraduate School of Paediatric Dentistry, Director: Prof. Patrizia Gallenzi Catholic University of Sacred Heart Rome Italy; ^4^ College of Dental Medicine ‐ Illinois Midwestern University Downers Grove Illinois USA

**Keywords:** autism spectrum disorder, awake bruxism, obstructive sleep apnea, sleep bruxism, validated questionnaires

## Abstract

Children with autism spectrum disorder (ASD) often present with sleep disorders, including obstructive sleep apnea (OSA), a condition characterized by upper airway obstruction during sleep. Bruxism has been recently described as being associated with OSA. This study aimed to assess the prevalence of OSA risk and bruxism in pediatric ASD patients compared to age and sex‐matched healthy controls using the validated screening tool Sleep‐Related Breathing Disorder scale of the Pediatric Sleep Questionnaire (SRBD‐PSQ). Fifty‐eight consecutive pediatric ASD patients were screened for OSA and bruxism at the Dentistry Unit of A. Gemelli Policlinic and compared to 58 healthy patients using chi‐square tests. Comparison between the two groups was repeated by controlling for body mass index (BMI) and behavioral symptoms with ANCOVA and logistic regression analyses. Of 58 ASD patients (10.3 ± 3.3 y/o, 74.5% males), 60.3% presented with an increased OSA risk, compared to 13.8% in the controls (*p* < 0.001, OR = 3.682, 95% CI: 1.933, 7.012). After controlling for BMI (which was significantly higher among ASD patients), those with ASD had significantly higher odds of OSA risk compared to controls (OR = 9.6, 95% CI: 3.56, 26.21). After controlling for the SRBD‐PSQ behavioral component, the association between ASD and OSA risk lost its significant difference (*p* < 0.862). No significant difference was found between ASD patients and controls in awake (3.6% vs. 6.9%, *p* = 0.680) and sleep (25.5% vs. 32.8%, *p* = 0.393) bruxism. Pediatric patients with ASD present at higher risk of OSA, most likely explained by the behavioral symptoms; self‐reported bruxism did not significantly differ compared to healthy controls.

## Summary

- Children with autism spectrum disorder present a higher risk of suffering from obstructive sleep apnea according to the Sleep‐Related Breathing Disorder scale of the Pediatric Sleep Questionnaire; however this result could be explained by the behavioral symptoms.
- Children with autism spectrum disorder do not report higher awake and/or sleep bruxism compared to peers without autism.

## 1 Introduction

Autism spectrum disorders (ASD) are defined as persistent deficits in social communication and interaction as well as restricted and repetitive patterns of behavior that impair daily functioning (American Psychiatric Association 2013); and present with a prevalence ranging from 1.7% to 2.5% among children and adolescents (Xu et al. 2018) and a male to female ratio of 4.4:1 (Scattoni et al. 2023). ASD diagnosis is made by a multifunctional team based on a comprehensive clinical evaluation from cognitive, communicative, social and emotional perspectives. Neurological and behavioral objective examinations are needed to rule out the presence of other pathologies that may cause clinical objectivity (Baird et al. 2003).

According to the American Psychiatric Association, ASD corresponds to neurodevelopmental disorders that affect social communication and behavioral routines. The Diagnostic and Statistical Manual of Mental Disorders (DSM‐5) unifies all categories of autism into a single diagnosis, with distinct levels of severity (American Psychiatric Association 2013).

Studies indicate that between 50% and 83% of individuals with ASD present sleep problems or disorders (Johnson et al. 2009; Kotagal and Broomall 2012; Ballester et al. 2020) with insomnia and circadian rhythm sleep–wake disorders being the most common complaints in this population (Souders et al. 2017; Ballester et al. 2020). Obstructive sleep apnea (OSA) is the most common sleep‐Related Breathing Disorder (SBD) and is characterized by recurrent episodes of complete or partial upper airway obstruction leading to reduced or absent breathing during sleep (Zaffanello et al. 2023).

Children with ASD have also been reported to present with higher rates of OSA. In fact, Hirata et al. (2016) reported a 28% prevalence of OSA in Japanese children aged 2–6 years with ASD compared to 15% in the general pediatric population; with higher rates of OSA in children with ASD being hypothesized to be attributable to increased rates of obesity and hypotonia in this population (Tomkies et al. 2019).

Some studies found an association between the two disorders so much so that OSA is thought to contribute to the clinical‐behavioral worsening of ASD patients (Hirata et al. 2016); in fact, behavioral problems were reported to significantly improve following adenotonsillectomy in children with both ASD and OSA (Murata et al. 2017); emphasizing the need for early diagnosis and treatment. Symptoms of pediatric OSA have been reported to overlap and exacerbate those of ASD, causing a delay in OSA diagnosis (Scattoni et al. 2023).

Therefore, it is crucial to implement sleep disorder screening at the earliest possible stage in children with ASD, in order to obtain a diagnosis and provide the most effective treatment to avoid a worsening of the clinical signs and symptoms of patients with ASD and concomitant OSA. Dental professionals have been requested to regularly screen both pediatric and adult patients for SBD in order to contribute to its early diagnosis and management (American Dental Association 2017; Burger 2017).

Bruxism is a repetitive jaw‐muscle activity characterized by clenching or grinding of the teeth and/or by bracing or trusting of the mandible. A temporal relationship between sleep bruxism (SB) and OSA has been suggested (Manfredini et al. 2015); however recent literature does not report a clear association between these conditions in the adult population (Błaszczyk et al. 2024), while it is still under discussion in the pediatric population (Pauletto et al. 2022). An increased risk of OSA and SB has been reported in pediatric patients with attention deficit and hyperactivity disorder (Alessandri‐Bonetti et al. 2024); and individuals with ASD have been described to be more likely to develop bruxism than controls (Granja et al. 2022); yet to the best of our knowledge, no studies so far have been conducted to observe OSA and SB risk on pediatric ASD subjects.

Aim of this study was to describe the prevalence of OSA risk and its association with bruxism in pediatric patients with ASD.

## 2 Methods

### 2.1 Study Design

The present study was a case–control cross‐sectional study, carried out at the Pediatric Dental Unit of a large university‐affiliated hospital. The study was approved by the Ethics Committee of the university where the study was conducted (ID 6270).

### 2.2 Eligibility Criteria

Participants were consecutive children who received a clinician‐based ASD diagnosis according to the DSM‐5 and referred to the clinic for dental evaluation. Inclusion criteria were pediatric patients (< 18 years of age) with an ASD diagnosis and whose patients/guardians were willing to sign an informed consent to participate in the study. Exclusion criteria were the presence of any other neurological disorder that could alter sleep architecture and/or those not willing to provide informed consent.

Because some studies have reported a relationship between sex, age and sleep problems in children (Krakowiak et al. 2008; Marcus et al. 2012), an equal number of age‐ and sex‐matched healthy controls were selected from all consecutive patients referred to the same Pediatric Dental Unit, after recruiting patients with ASD.

### 2.3 Study Procedures

As part of routine care, patients undergo a complete dental examination and interview, and their parents/guardians are asked to fill out the SBD scale of the Pediatric Sleep Questionnaire (SRBD‐PSQ, see Outcome Measures) (Chervin et al. 2000). All patients with high OSA risk based on the SRBD‐PSQ were referred to a sleep center to obtain an OSA definite diagnosis through polysomnographic (PSG) recording.

### 2.4 Outcome Measures

The following outcome measures were collected in this study.

Risk of OSA was calculated based on the SRBD‐PSQ. The SRBD‐PSQ is a rapid and highly sensitive screening tool for pediatric OSA, widely used in research and clinical settings for sleep problems in children (Incerti Parenti et al. 2021). It consisted of 22 questions, divided into three sections based on nocturnal, diurnal, and cognitive symptoms. The questionnaire had an answer of “yes” (which was assigned 1 point on the rating scale) or “no/do not know” (0 points on the rating scale). According to published scoring criteria, a total score was calculated by dividing the number of “yes” by the number of “yes” plus “no,” while the “do not know” answers were excluded from the calculation (Chervin et al. 2000). The final value is comprised between 0 and 1, with scores > 0.33 identifying an increased risk of OSA with a sensitivity of 85% and specificity of 87% within the general pediatric population (Freezer et al. 1995).

Considering that cognitive symptoms in children with ASD could potentially be due to the ASD diagnosis rather than to OSA, the two cohorts of participants were also compared on the cognitive behavior section of the SRBD‐PSQ (i.e., last six questions of the questionnaire).

### 2.5 Demographics, Including Age and Sex

Presence of probable awake and SB was evaluated based on self‐report (by parents/caregivers' report) and clinical examination (by assessing the presence of wear facets), according to the international consensus on bruxism (Lobbezoo et al. 2013).

Weight status of each child was assessed by computing the body mass index (BMI), and interpreted as underweight, normal‐weight, overweight, and obese using Cole et al.'s (2000, 2007) cut‐off points in pediatric populations.

### 2.6 Statistical Analysis

Demographic data were analyzed by descriptive statistics and reported as mean ± standard deviation, or as absolute frequencies and percentages if related to categorical variables. The proportions of patients at risk of OSA (i.e., SRBD‐PSQ ≥ 0.33) and with probable bruxism among participants with ASD and controls were compared with chi‐square tests. Odds ratios (OR) and 95% confidence intervals (CI) were calculated. Given their influence on the risk of OSA, the two groups were compared in total value of the SRBD‐PSQ (as continuous data) by controlling for BMI and the SRBD‐PSQ cognitive behavior component with analysis of covariance (ANCOVA). The comparison was repeated by controlling for BMI and for behavioral components with logistic regression analysis. Statistical analyses were conducted with SPSS (IBM SPSS Statistics, v. 29, IBM Corp., Armonk, NY), by setting the significance level at 0.05.

## 3 Results

All 116 eligible patients (being 58 participants with ASD and 58 age and sex‐matched controls, mean age 10.4 ± 3.1 y/o, 81.0% males) accepted to participate in the study. No statistically significant difference was found in demographics between children with ASD (mean age 10.8 ± 3.0 y/o, 81.0% males) and controls (mean age 10.0 ± 3.1 y/o, 81.0% males). All included patients were white Caucasian.

Based on the results of the SRBD‐PSQ assessment, 35 (60.3%) participants with ASD exhibited an increased risk of OSA compared to 8 (13.8%) of the control group; thus demonstrating a statistically significant difference between the two groups (*p* < 0.001, Cramer's *V* = 0.48, OR = 3.682, 95% CI 1.933, 7.012) (Table 1).

**TABLE 1:** Comparison of SRBD‐PSQ outcomes between the ASD and control groups.

Outcome	ASD (%)	Control (%)	*χ* ^2^	*p*
OSA risk	60.3	13.8	29.5	< 0.001
Sleep bruxism	25.5	32.8	0.73	0.393
Awake bruxism	3.6	6.9	0.17	0.680

Abbreviation: *χ* ^2^, chi‐square score.

Given that participants with ASD had significantly greater BMI than controls (23.5 ± 6.6 vs. 18.5 ± 4.1, *p* < 0.001, Cohen's *d* = 0.92), a logistic regression was performed to ascertain the effects of group belonging (ASD or control) and BMI on the likelihood that participants had higher risk of OSA. The model explained 31.9% (Nagelkerke *R* ^2^) of the variance in risk of OSA and correctly classified 74.8% of the cases. After controlling for BMI, individuals with ASD still had significantly higher odds of being at risk for OSA compared to controls (*p* < 0.001). ASD participants exhibited 9.6 times higher odds of being at risk for OSA than controls (95% CI: 3.56, 26.21). Conversely, after controlling for diagnostic group (ASD vs. control), BMI was not a significant predictor of OSA risk (*p* = 0.731). For each one‐unit increase in BMI, the odds of being at risk for OSA increased by 1.4%, but this change was not statistically significant (Table 2).

**TABLE 2:** Logistic regression assessing the effects of group belonging (ASD vs. control) and BMI on the likelihood of higher OSA risk.

Predictor	*B*	SE	Wald X	*p*	OR	95% CI
BMI	0.014	0.040	0.119	0.731	1.014	0.938, 1.096
ASD group	2.268	0.510	19.807	< 0.001	9.656	3.556, 26.212

Abbreviations: *B*, standardized regression coefficient; CI, confidence interval; OR, odds ratio; SE, standard error.

As expected, participants with ASD exhibited significantly higher cognitive symptoms compared to controls (0.8 ± 0.3 vs. 0.1 ± 0.2, *p* < 0.001, Cohen's *d* = 2.32). To account for potential confounding by behavioral symptoms, a logistic regression model was conducted with SRBD‐PSQ cognitive behavior section score as a covariate. After adjusting for cognitive behavior, the association between ASD and OSA risk lost its significant difference (*p* = 0.862, OR = 1.13, 95% CI 0.28, 4.69), suggesting that the increased risk of OSA in the ASD group may be partly explained by higher cognitive/behavioral symptoms in ASD patients (Table 3).

**TABLE 3:** Logistic regression assessing the effects of cognitive symptoms and group belonging (ASD vs. control) on the likelihood of higher OSA risk.

Predictor	*B*	SE	Wald	*p*	OR	95% CI
Cognitive symptoms	4.664	1.083	18.559	< 0.001	106.06	12.71, 885.37
ASD group	0.126	0.724	0.030	0.862	1.13	0.28, 4.69

Abbreviations: *B*, standardized regression coefficient; CI, confidence interval; OR, odds ratio; SE, standard error.

Finally, a one‐way ANCOVA was conducted to examine group differences in SRBD‐PSQ total score while controlling for BMI and cognitive behavioral symptoms (PSQ section 3). The analysis revealed a significant effect of cognitive behavioral symptoms ($F_{\left(1,107\right)}$ = 45.122, *p* < 0.001), but not for group (*p* = 0.046) or BMI (*p* = 0.132), suggesting that behavioral symptoms explain the elevated OSA risk scores observed in children with ASD (Table 4).

**TABLE 4:** ANCOVA results examining the effects of group (ASD vs. control), BMI, and cognitive symptoms on SRBD‐PSQ total scores.

Predictor	$F_{\left(1,107\right)}$	*p*	Note
Group (ASD vs. control)	4.08	0.046	Not significant after controlling for covariates
BMI	2.30	0.132	Not significant
Cognitive symptoms	45.122	< 0.001	Significant effect on SRBD‐PSQ total score

Abbreviations: B, standardized regression coefficient; CI, confidence interval; OR, odds ratio; SE, standard error.

As for definite OSA diagnosis, the first eight children with ASD presenting high risk of OSA were referred to the sleep physician for PSG evaluation; however none of them was able to undergo a sleep study due to lack of compliance; therefore the decision was made not to continue referring patients for additional PSG examination.

As for probable bruxism, there were no statistically significant differences in SB (25.5% among ASD vs. 32.8% among controls, *p* = 0.393, Cramer's *V* = 0.08) nor in awake bruxism (3.6% among ASD vs. 6.9% among controls, *p* = 0.680, Cramer's *V* = 0.07) between the two groups (Table 1).

Probable sleep and awake bruxism were also not associated with OSA risk in either ASD children or controls (all *p*'s > 0.05).

## 4 Discussion

The present study aimed to assess the prevalence of OSA risk and probable bruxism in pediatric patients with ASD compared to age‐ and sex‐matched healthy controls. A significantly higher risk of OSA was observed in children with ASD compared to controls. This result is consistent with what is reported in the available literature that indicates sleep problems, including OSA, to be more common in children with ASD than controls when evaluated by questionnaires (Hirata et al. 2016). However, despite children with ASD presenting more symptoms of OSA, a recent study reported no statistically significant differences between children with and without ASD in total apnea‐hypopnea index and obstructive apnea‐hypopnea index, derived from PSG (Santapuram et al. 2022). This can explain our finding that OSA risk obtained from SRBD‐PSQ seems to be significantly influenced by answers obtained in the behavioral component. Since symptoms of ASD may overlap with those of OSA, distinguishing between these conditions may represent an additional challenge and lead to delays in OSA diagnosis and management (Santapuram et al. 2022). In our sample, OSA confirmation through PSG was not obtained due to the lack of compliance of children with ASD in undergoing PSG, suggesting that other diagnostic methods should be considered in this population. However, due to the high impact of the behavioral component of the SRBD‐PSQ on the number of patients considered at risk of OSA, it can be hypothesized that SRBD‐PSQ may not be the appropriate tool for OSA screening in children with ASD either. Other screening/diagnostic methods for OSA such as pulse oximetry (Incerti Parenti et al. 2024) could be considered to better assist in OSA screening and diagnosis in this population.

In fact, the impact of OSA on behavioral problems in this population is still unclear, but previous studies have suggested that the presence of OSA may be responsible for worsening signs and symptoms of ASD (Ramanathan et al. 2023), and behavioral problems have been reported to improve following adenotonsillectomy in children with ASD and OSA (Murata et al. 2017). Together, this suggests that OSA may have a higher symptom burden in this population and reinforces the importance of sleep screening in all individuals with ASD. Future studies are needed to assess whether early diagnosis and treatment of OSA could improve ASD symptoms.

A 10% OSA risk in healthy children as observed in the present sample is consistent with what is reported in the available literature (Santapuram et al. 2022), therefore corroborating the present results. The sample analyzed consisted of a higher prevalence of the male component, which is not surprising considering the higher male‐to‐female ratio that characterizes ASD (Loomes et al. 2017).

Children with ASD were found to have greater BMI than controls; however BMI was not a significant predictor of OSA in this sample. This is in contrast with what is reported in a recent study suggesting that obesity is a predictor of severe OSA in children (Tomkies et al. 2019); however, the study by Tomkies et al. was conducted on a smaller sample and with patients of different ethnicity, which can possibly differ from those examined in the present sample. Moreover, our study focused on patients at high risk and not on confirmed OSA diagnosis, which could also explain discrepancies in the findings.

As for probable bruxism, no differences were found between ASD and controls regarding awake or SB, and no association was found between bruxism and OSA risk in the sample analyzed. Additionally, recent findings by Costa‐Silva et al. (2025) explored the relationship between SB and SBDs in children and adolescents with ASD, highlighting how these comorbidities may be interconnected and suggesting that future studies should further investigate this overlap. This is partially in contrast with what was reported by Granja et al. (2022), however authors also mentioned that the quality of evidence was very low and therefore results were still uncertain.

The present study has some limitations; the major one being the lack of a PSG to confirm the OSA diagnosis. However, since no compliance could be obtained from children with ASD undergoing PSG, the decision was made to discontinue referring children for PSG evaluation. Another limitation is relatively small.

Despite these limitations, the present study is the first one to assess OSA risk in pediatric patients with ASD according to the SRBD‐PSQ, and to further explore its use.

Based on the present study we strongly suggest pediatric dentists provide OSA screening in all patients as recommended by the American Academy of Pediatrics (Erichsen et al. 2012); however, we highlight the need for better assessment tools for both OSA screening and diagnosis in children with ASD, given the potential overlap between OSA and ASD on cognitive behavioral symptoms. In conclusion, children with ASD presented a higher risk of OSA compared to age‐ and sex‐matched controls without ASD according to SRBD‐PSQ; however, these results were influenced by the cognitive behavioral symptoms of these patients. New screening tools specific for pediatric patients with ASD are needed. Awake and SB were not found to be more common in children with ASD compared to controls.

## Conflicts of Interest

The authors declare no conflicts of interest.

## Data Availability

The data that support the findings of this study are available on request from the corresponding author. The data are not publicly available due to privacy or ethical restrictions.

## References

[aur70149-bib-0001] Alessandri‐Bonetti, A. , F. Guglielmi , G. Deledda , L. Sangalli , C. Brogna , and P. Gallenzi . 2024. “Malocclusions, Sleep Bruxism, and Obstructive Sleep Apnea Risk in Pediatric ADHD Patients: A Prospective Study.” Journal of Attention Disorders 28, no. 6: 1017–1023. 38327066 10.1177/10870547231226139

[aur70149-bib-0002] American Dental Association . 2017. The Role of Dentistry in the Treatment of Sleep Related Breathing Disorders. American Dental Association.

[aur70149-bib-0003] American Psychiatric Association . 2013. Diagnostic and Statistical Manual of Mental Disorders. 5th ed. Author.

[aur70149-bib-0004] Baird, G. , H. Cass , and V. Slonims . 2003. “Diagnosis of Autism.” BMJ 327, no. 7413: 488–493. 12946972 10.1136/bmj.327.7413.488 PMC188387

[aur70149-bib-0005] Ballester, P. , A. L. Richdale , E. K. Baker , and A. M. Peiró . 2020. “Sleep in Autism: A Biomolecular Approach to Aetiology and Treatment.” Sleep Medicine Reviews 54: 101357. 32759030 10.1016/j.smrv.2020.101357

[aur70149-bib-0006] Błaszczyk, B. , M. Waliszewska‐Prosół , M. Więckiewicz , et al. 2024. “Sleep Bruxism (SB) May Be Not Associated With Obstructive Sleep Apnea (OSA): A Comprehensive Assessment Employing a Systematic Review and Meta‐Analysis.” Sleep Medicine Reviews 78: 101994. 39182463 10.1016/j.smrv.2024.101994

[aur70149-bib-0008] Burger, D. 2017. Sleep‐Related Breathing Disorder Treatment Outlined in New Policy. ADA News.

[aur70149-bib-0009] Chervin, R. D. , K. Hedger , J. E. Dillon , and K. J. Pituch . 2000. “Pediatric Sleep Questionnaire (PSQ): Validity and Reliability of Scales for Sleep‐Disordered Breathing, Snoring, Sleepiness, and Behavioral Problems.” Sleep Medicine 1, no. 1: 21–32. 10733617 10.1016/s1389-9457(99)00009-x

[aur70149-bib-0010] Cole, T. J. , M. C. Bellizzi , K. M. Flegal , and W. H. Dietz . 2000. “Establishing a Standard Definition for Child Overweight and Obesity Worldwide: International Survey.” BMJ 320: 1240–1243. 10797032 10.1136/bmj.320.7244.1240 PMC27365

[aur70149-bib-0011] Cole, T. J. , K. M. Flegal , D. Nicholls , and A. A. Jackson . 2007. “Body Mass Index Cut Offs to Define Thinness in Children and Adolescents: International Survey.” BMJ 335: 194. 17591624 10.1136/bmj.39238.399444.55 PMC1934447

[aur70149-bib-0012] Costa‐Silva, J. G. V. , S. M. Paiva , F. Vargas‐Ferreira , J. M. C. Serra‐Negra , and R. G. Vieira‐Andrade . 2025. “Possible Sleep Bruxism in Children and Adolescents With Autism Spectrum Disorder: Association With Parental Stress and Sleep Disorders.” Journal of Autism and Developmental Disorders 55: 4372–4379. 40024969 10.1007/s10803-025-06763-6

[aur70149-bib-0013] Erichsen, D. , C. Godoy , F. Gränse , J. Axelsson , D. Rubin , and D. Gozal . 2012. “Screening for Sleep Disorders in Pediatric Primary Care: Are We There Yet?” Clinical Pediatrics 51, no. 12: 1125–1129. 23203980 10.1177/0009922812464548

[aur70149-bib-0014] Freezer, N. J. , I. K. Bucens , and C. F. Robertson . 1995. “Obstructive Sleep Apnoea Presenting as Failure to Thrive in Infancy.” Journal of Paediatrics and Child Health 31, no. 3: 172–175. 7669373 10.1111/j.1440-1754.1995.tb00779.x

[aur70149-bib-0015] Granja, G. L. , J. T. Lacerda‐Santos , R. T. Firmino , et al. 2022. “Occurrence of Bruxism in Individuals With Autism Spectrum Disorder: A Systematic Review and Meta‐Analysis.” Special Care in Dentistry 42, no. 5: 476–485. 35263459 10.1111/scd.12707

[aur70149-bib-0016] Hirata, I. , I. Mohri , K. Kato‐Nishimura , et al. 2016. “Sleep Problems Are More Frequent and Associated With Problematic Behaviors in Preschoolers With Autism Spectrum Disorder.” Research in Developmental Disabilities 49–50: 86–99. 10.1016/j.ridd.2015.11.002 26672680

[aur70149-bib-0017] Incerti Parenti, S. , M. L. Bartolucci , A. Fiordelli , P. Gigola , C. Paganelli , and G. Alessandri‐Bonetti . 2024. “The Diagnostic Accuracy of Overnight Oximetry for Pediatric Obstructive Sleep Apnea: A Systematic Review and Meta‐Analysis.” Applied Sciences 14: 10208.

[aur70149-bib-0018] Incerti Parenti, S. , A. Fiordelli , M. L. Bartolucci , S. Martina , V. D'Antò , and G. Alessandri‐Bonetti . 2021. “Diagnostic Accuracy of Screening Questionnaires for Obstructive Sleep Apnea in Children: A Systematic Review and Meta‐Analysis.” Sleep Medicine Reviews 57: 101464. 33827032 10.1016/j.smrv.2021.101464

[aur70149-bib-0019] Johnson, K. P. , F. Giannotti , and F. Cortesi . 2009. “Sleep Patterns in Autism Spectrum Disorders.” Child and Adolescent Psychiatric Clinics of North America 18, no. 4: 917–928. 19836696 10.1016/j.chc.2009.04.001

[aur70149-bib-0021] Kotagal, S. , and E. Broomall . 2012. “Sleep in Children With Autism Spectrum Disorder.” Pediatric Neurology 47, no. 4: 242–251. 22964437 10.1016/j.pediatrneurol.2012.05.007

[aur70149-bib-0022] Krakowiak, P. , B. Goodlin‐Jones , I. Hertz‐Picciotto , L. A. Croen , and R. L. Hansen . 2008. “Sleep Problems in Children With Autism Spectrum Disorders, Developmental Delays, and Typical Development: A Population‐Based Study.” Journal of Sleep Research 17, no. 2: 197–206. 18482108 10.1111/j.1365-2869.2008.00650.x PMC4041696

[aur70149-bib-0023] Lobbezoo, F. , J. Ahlberg , A. G. Glaros , et al. 2013. “Bruxism Defined and Graded: An International Consensus.” Journal of Oral Rehabilitation 40, no. 1: 2–4. 23121262 10.1111/joor.12011

[aur70149-bib-0024] Loomes, R. , L. Hull , and W. P. L. Mandy . 2017. “What Is the Male‐to‐Female Ratio in Autism Spectrum Disorder? A Systematic Review and Meta‐Analysis.” Journal of the American Academy of Child & Adolescent Psychiatry 56, no. 6: 466–474. 28545751 10.1016/j.jaac.2017.03.013

[aur70149-bib-0025] Manfredini, D. , L. Guarda‐Nardini , R. Marchese‐Ragona , and F. Lobbezoo . 2015. “Theories on Possible Temporal Relationships Between Sleep Bruxism and Obstructive Sleep Apnea Events: An Expert Opinion.” Schlaf & Atmung 19, no. 4: 1459–1465. 25794544 10.1007/s11325-015-1163-5

[aur70149-bib-0026] Marcus, C. L. , L. J. Brooks , K. A. Draper , et al. 2012. “Diagnosis and Management of Childhood Obstructive Sleep Apnea Syndrome.” Pediatrics 130, no. 3: e714–e755. 22926176 10.1542/peds.2012-1672

[aur70149-bib-0027] Murata, E. , I. Mohri , K. Kato‐Nishimura , et al. 2017. “Evaluation of Behavioral Change After Adenotonsillectomy for Obstructive Sleep Apnea in Children With Autism Spectrum Disorder.” Research in Developmental Disabilities 65: 127–139. 28514706 10.1016/j.ridd.2017.04.012

[aur70149-bib-0028] Pauletto, P. , H. Polmann , J. Conti Réus , et al. 2022. “Sleep Bruxism and Obstructive Sleep Apnea: Association, Causality or Spurious Finding? A Scoping Review.” Sleep 45, no. 7: zsac073. 35443064 10.1093/sleep/zsac073

[aur70149-bib-0029] Ramanathan, D. , P. Kipnis , P. Klaas , K. A. Aaron , and S. Anne . 2023. “Attention‐Deficit/Hyperactivity Disorder in Children With Hearing Loss.” International Journal of Pediatric Otorhinolaryngology 164: 111191. 10.1016/j.ijporl.2023.111612 37329702

[aur70149-bib-0030] Santapuram, P. , H. Chen , A. S. Weitlauf , M. O. A. Ghani , and A. S. Whigham . 2022. “Investigating Differences in Symptomatology and Age at Diagnosis of Obstructive Sleep Apnea in Children With and Without Autism.” International Journal of Pediatric Otorhinolaryngology 158: 111191. 35636082 10.1016/j.ijporl.2022.111191

[aur70149-bib-0031] Scattoni, M. L. , L. M. Fatta , M. Micai , et al. 2023. “Autism Spectrum Disorder Prevalence in Italy: A Nationwide Study Promoted by the Ministry of Health.” Child and Adolescent Psychiatry and Mental Health 17: 125. 37898807 10.1186/s13034-023-00673-0 PMC10613370

[aur70149-bib-0032] Souders, M. C. , S. Zavodny , W. Eriksen , et al. 2017. “Sleep in Children With Autism Spectrum Disorder.” Current Psychiatry Reports 19, no. 6: 34. 28502070 10.1007/s11920-017-0782-x PMC5846201

[aur70149-bib-0033] Tomkies, A. , R. F. Johnson , G. Shah , M. Caraballo , P. Evans , and R. B. Mitchell . 2019. “Obstructive Sleep Apnea in Children With Autism.” Journal of Clinical Sleep Medicine 15, no. 10: 1469–1476. 31596212 10.5664/jcsm.7978 PMC6778353

[aur70149-bib-0035] Xu, G. , L. Strathearn , B. Lui , and W. Bao . 2018. “Prevalence of Autism Spectrum Disorder Among US Children and Adolescents, 2014–2016.” JAMA 319, no. 1: 81–82. 29297068 10.1001/jama.2017.17812 PMC5833544

[aur70149-bib-0036] Zaffanello, M. , G. Piacentini , L. Nosetti , and L. Zoccante . 2023. “Sleep Disordered Breathing in Children With Autism Spectrum Disorder: An In‐Depth Review of Correlations and Complexities.” Children (Basel) 10, no. 10: 1609. 37892271 10.3390/children10101609 PMC10605434

